# ParaPET: non-invasive deep learning method for direct parametric brain PET reconstruction using histoimages

**DOI:** 10.1186/s13550-024-01072-y

**Published:** 2024-01-30

**Authors:** Rajat Vashistha, Hamed Moradi, Amanda Hammond, Kieran O’Brien, Axel Rominger, Hasan Sari, Kuangyu Shi, Viktor Vegh, David Reutens

**Affiliations:** 1 Centre for Advanced Imaging, University of Queensland, Brisbane, Australia; 2 ARC Training Centre for Innovation in Biomedical Imaging Technology, University of Queensland, Brisbane, Australia; 3 Siemens Healthcare Pty Ltd, Melbourne, Australia; 4 Advanced Clinical Imaging Technology, Siemens Healthcare AG, Lausanne, Switzerland; 5 Department of Nuclear Medicine, Inselspital, Bern University Hospital, University of Bern, Freiburgstrasse 18, 3010 Bern, Switzerland

**Keywords:** Positron emission tomography, Direct parametric image reconstruction, Deep learning, Histoimages

## Abstract

**Background:**

The indirect method for generating parametric images in positron emission tomography (PET) involves the acquisition and reconstruction of dynamic images and temporal modelling of tissue activity given a measured arterial input function. This approach is not robust, as noise in each dynamic image leads to a degradation in parameter estimation. Direct methods incorporate into the image reconstruction step both the kinetic and noise models, leading to improved parametric images. These methods require extensive computational time and large computing resources. Machine learning methods have demonstrated significant potential in overcoming these challenges. But they are limited by the requirement of a paired training dataset. A further challenge within the existing framework is the use of state-of-the-art arterial input function estimation via temporal arterial blood sampling, which is an invasive procedure, or an additional magnetic resonance imaging (MRI) scan for selecting a region where arterial blood signal can be measured from the PET image. We propose a novel machine learning approach for reconstructing high-quality parametric brain images from histoimages produced from time-of-flight PET data without requiring invasive arterial sampling, an MRI scan, or paired training data from standard field-of-view scanners.

**Result:**

The proposed is tested on a simulated phantom and five oncological subjects undergoing an 18F-FDG-PET scan of the brain using Siemens Biograph Vision Quadra. Kinetic parameters set in the brain phantom correlated strongly with the estimated parameters (*K*_1_*, k*_2_ and *k*_3_, Pearson correlation coefficient of 0.91, 0.92 and 0.93) and a mean squared error of less than 0.0004. In addition, our method significantly outperforms (*p *< 0.05, paired t-test) the conventional nonlinear least squares method in terms of contrast-to-noise ratio. At last, the proposed method was found to be 37% faster than the conventional method.

**Conclusion:**

We proposed a direct non-invasive DL-based reconstruction method and produced high-quality parametric maps of the brain. The use of histoimages holds promising potential for enhancing the estimation of parametric images, an area that has not been extensively explored thus far. The proposed method can be applied to subject-specific dynamic PET data alone.

**Supplementary Information:**

The online version contains supplementary material available at 10.1186/s13550-024-01072-y.

## Introduction

Dynamic PET imaging captures the spatiotemporal distribution of a radiotracer inside the body in a four-dimension, three dimensions in space and one dimension in time [1]. Parametric images are generated by utilizing a kinetic model based on the temporal uptake of the tracer [2, 3]. The selection of tracer is application specific; for example, the ^18^F-fluorodeoxyglucose (^18^F-FDG) is used in oncology to study glucose metabolism in tumour [4, 5]. The estimated kinetic parameters provide valuable insights into the physiological and biochemical processes occurring within the tissue. These parameters have been widely used in research applications and are increasingly being explored for clinical diagnosis, prognosis, and treatment planning. ^18^F-FDG-PET parametric imaging is effective in monitoring brain disorders [6]. In addition, kinetic parameters play an important role in research investigations identifying receptor-binding agents*;* for example, binding potential and distribution volume are used for in vivo* imaging of* neuroinflammation and dopamine receptor imaging [7]. However, the accurate assessment of the underlying disease state relies on the accuracy of parametric images. Parametric images are adversely affected by noise in dynamic imaging data.

The conventional approach for calculating parametric images in PET requires the reconstruction of dynamic images from the projection data, followed by a voxel-wise fitting of tissue activity curves and measurement of the arterial input function [2, 3]. This indirect method is prone to producing low signal-to-noise ratio parametric images due to the difficulty in modelling noise in the image space [8]. On the other hand, direct methods of reconstruction transform parametric images from the raw projection data in a single step and provide improved noise modelling capabilities [9–11]. However, the convergence of algorithms associated with direct methods is computationally intensive and time-consuming, necessitating the need for offline computation.

A further challenge within the existing framework is the use of state-of-the-art arterial input function estimation via temporal arterial blood sampling, an invasive procedure, or involves an additional magnetic resonance imaging (MRI) scan for selecting a region where arterial blood signal can be measured from the PET image [12, 13]. Alternative approaches that do not require an extra MRI for region-of-interest selection are valuable, but they tend to be time-intensive, relying on the manual inspection of PET data to segment and mask the carotid artery [14]. In addition, population-based input functions have shown promising capabilities, particularly in the context of accurate Patlak analysis for shortened dynamic acquisitions [15]. These limitations warrant the development of a new method that should accurately reconstruct parametric images with a high signal-to-noise ratio in a shorter duration, specific to the individual and non-invasively estimates the arterial input function from the same images as those used for parametric image creation.

Deep learning (DL) methods have demonstrated significant potential in enhancing image quality by addressing these challenges. Typically, the network training in these studies relies on supervised learning with high-quality training labels, which are often challenging to obtain in clinical practice. Recent methods have focused on unsupervised approaches for indirect and direct parametric methods, but necessities acquiring corresponding anatomical images, such as CT or MRI [10, 16]. It is also important to note that these methods are only limited to parametric models assuming a linear relationship between the tracer concentration and time. In addition, the deep learning-based direct parametric method operates on projection data (sinogram), necessitating neural networks to undertake computationally intensive memory-space operations to transform the data into the image domain.

Modern time-of-flight (TOF) PET scanner provides better timing resolution by including the arrival time difference between a pair of detected photons. This information is used to correct position estimation of emission along the line of response [17–19]. Histoprojection takes advantage of the reduced position uncertainty resulting from TOF correction to improve image reconstruction. It accomplishes this by histogramming coincidence events into image space [20]. A histoprojection can be considered as a blurred version of the final image along the TOF dimension. By combining histoprojections from multiple views, a histoimage is generated [20]. The transformed representation of the raw measurement data as histoimage is well suited for deep learning due to its local correlation and the fact that reconstruction can be considered as an image-to-image operation.

Additionally, histoimages have been used previously to achieve faster PET image reconstructions [21]. Here, uncertainty in the time difference calculation for every projection results in inaccurate position estimation that can be modelled as a 1-D Gaussian convolution in the direction of the line of response [17, 21]. Consequently, the transformed raw data obtained from the scanner is susceptible to blurring along the TOF dimension. The use of histoimages holds promising potential for enhancing the estimation of parametric images, an area that has not been extensively explored thus far. The application of neural networks designed for image deblurring and denoising is ubiquitous with general deep learning research and particularly in the field of medical imaging [22].

## Methods

**Fig. 1:**
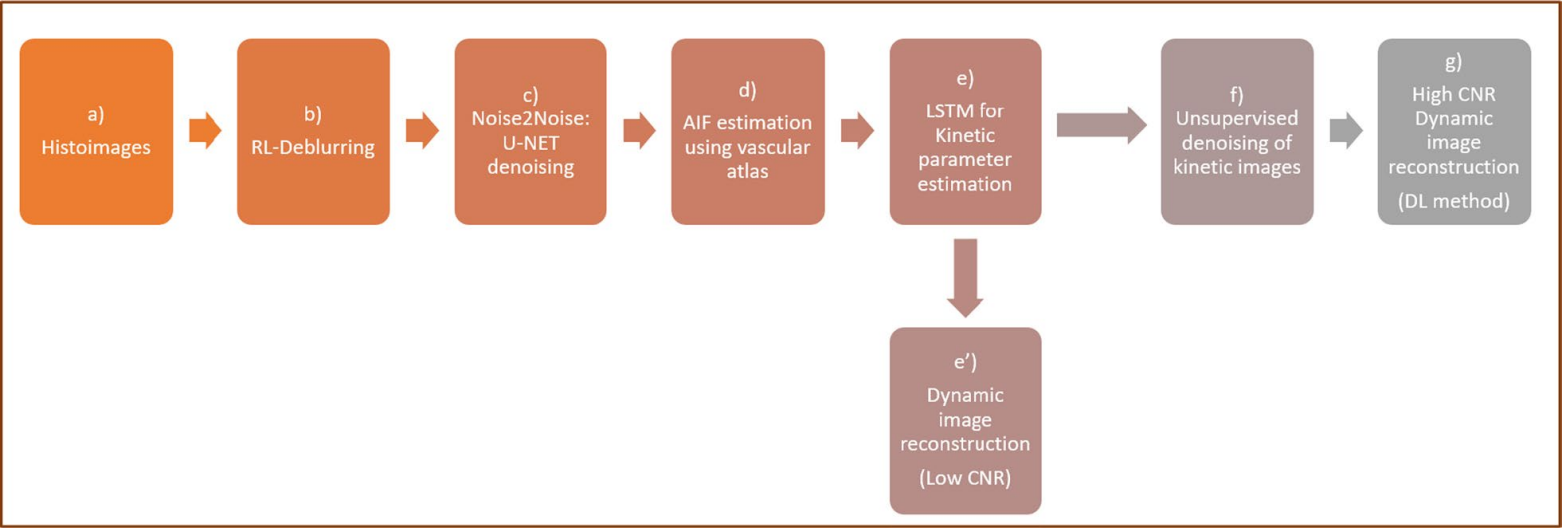
The direct parametric image reconstruction pipeline. **a** Histoimages are deblurred using Richardson-lucy delurring algorithm (**b**). Deep learning techniques are employed at steps **c** denoising without clean target, **e** long short-term memory cells (LSTM), **f** deep image prior, and while step **g** involves the reconstruction of dynamic images from denoised parametric images to validate the method. Step **d** involves the estimation of the arterial input function

We propose a two-step approach for deblurring histoimages inspired by the work of Lehtinen et al. [23]. The first step involves the use of deconvolution to deblur an image, followed by a denoising step. The denoising process initially involves the training of a U-NET network to handle different levels of Poisson noise. Subsequently, a 1D-LSTM model trained using simulated tissue time activity curves (generated using conventional compartmental modelling) is applied to calculate pixel-wise kinetic parameters. After training, the 1D-LSTM model was used with the denoised images and the AIF estimated from brain images to generate images of the kinetic parameters. Finally, an unsupervised deep image prior was applied to enhance the parametric images and reconstruct the individual dynamic images [24]. The image reconstruction pipeline is provided in Fig. 1. The method is tested in five subjects undergoing an ^18^F-FDG-PET scan of the brain.

### Human data and histoimages

**Fig. 2:**
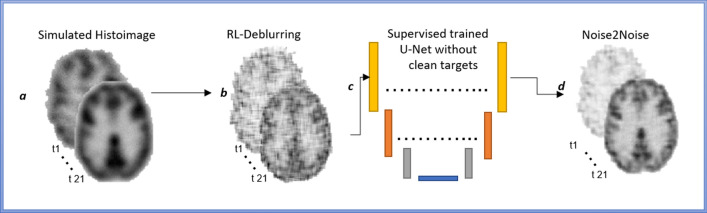
Detail of the first three steps in Fig. 1 (i.e. a–c) showing how histomimages were deblurred and then cleaned using a U-Net for an example subject. Histoimages in **a** were generated using a Gaussian filter as described in the methods section, followed by iterative deconvolution via the Richardson-Lucy algorithm (**b**). The U-Net architecture used to denoise images is showcased in **c**, resulting in dynamic denoised images as shown in **d**

Data were obtained in collaboration in accordance with a previous study [25]. The human total body PET data was acquired using a Biograph Vision Quadra in list mode started 15 s prior to the intravenous injection of ^18^F-FDG (mean activity: 235 ± 51 MBq; i.e. approximately 3 MBq/kg). Emission data were acquired for 65 min and binned over 62 frames: 2 × 10 s, 30 × 2 s, 4 × 10 s, 8 × 30 s, 4 × 60 s, 5 × 120 s, and 9 × 300 s [25]. Images were reconstructed using the Siemens OSEM-PSF + TOF reconstruction algorithm with conventional correction strategies for attenuation, scatter, and random coincidences [25]. This study involved already-acquired data from a Total Body PET scanner, and the theoretical definition of histoimages was used to generate a simulated version from true dynamic PET images [17]. The adopted methodology involves the generation of histoimages using a Gaussian filter (Eq. 1), as shown in Fig. 2a. The standard deviation (${\sigma }_{{\text{TOF}}}$) of the filter represents the blurring caused by the TOF-PET scanner. Finally, the blurred histoimage was represented as the convolution of the clean dynamic PET image with the kernel plus added Poisson noise (*η*) and the general image reconstruction problem was converted into a deblurring problem:

1 $${\text{Blurring}}_{{{\text{TOF}}}} = \left( {\frac{{{\text{Gauss}}\left( {x,y,\sqrt {2\sigma_{{{\text{TOF}}}} } } \right)}}{{\sqrt {x^{2} + y^{2} } }} + \eta } \right),$$

### Deconvolution and denoising

The deconvolution of histoimages was performed using non-blind iterative [26] and non-iterative [27] algorithms. Richardson–Lucy deconvolution (RL–Deconv) was chosen for iterative deconvolution (Fig. 2b) [28], and the Wiener filter was used for non-iterative deconvolution. The kernel for RL–deblurring was ${\sigma }_{{\text{TOF}}}$ as defined by the TOF-PET scanner. The non-blind deblurring technique was adopted since the blurring kernel for the TOF-PET scanner is known.

Among these algorithms, non-iterative methods are the fastest to execute but do not provide optimal image quality, particularly in the presence of noise. This is of particular relevance for PET histoimages degraded by Poisson noise. Iterative deblurring algorithms, such as the Richardson-Lucy deconvolution, approximate a clean image from the blurred counterpart when dealing with images that have complex or variable blurring characteristics. However, for high-noise images, the Richardson-Lucy algorithm adds significant levels of coloured noise to the solution (see Fig. 2b) [29].

Deep learning methods are suitable for different denoising problems, but largely require pairing of noisy and clean images for training. Extending on the work by Lehtinen et al. [23, 29], we decompose the deblurring of histoimages into a denoising problem and trained a network without having clean target images. Here, we apply the Richardson–Lucy algorithm (with early stopping at 30 epochs) to deblur atlas-based images with varying magnitude of Poisson noise, resulting in paired images of different noise levels [28, 30]. The underlying concept of this approach is that a neural network, trained with numerous sets of noisy images of a specific clean scene, can approximate the expected loss between the network output and the corresponding clean target, by minimizing the expected loss between the network output and the noisy target. Figure 2c depicts the U-NET used to perform this task. Sixty epochs on 650 atlas images were used to train the U-NET using mean squared error loss function. Data augmentation involving translation, scaling, rotation, and transformation was performed over the standard atlas before creating paired images.

### Simulated noised TAC and AIF

In dynamic PET, the measured tissue signal is the sum of radioactivity signal compartments, each of which follow an exponential behaviour. The two-tissue compartment model (2-TCM) for the ^18^F-FDG-PET tracer is used to simulate the total tissue impulse response function.

The total concentration (*C*_T_ (t)) equation describes ^18^F-FDG kinetics in the brain for irreversible 2-TCM, where *K*_1_, *k*_2_ and *k*_3_ are the rate constants and can be derived from the impulse response function variables* (w*’s and *v*’s (see Eqs. 2, 3 and 4) [31]). Additionally, Cp(t) is the plasma input function and Vb is the vascular blood volume. Feng’s arterial input function formula [31] was used to simulate Cp’s, where the parameters *A*_1_ (25,900 to 3700), *A*_2_ (999 to 740), *A*_3_ (814 to 666), *L*_1_ (− 5.0 to − 3.5), *L*_2_ (− 2.0 to − 0.1), *L*_3_ (− 0.0104 to − 0.0190) have known ranges in units of ${\text{KBq}}/{\text{ml}}/{\text{min}}$ for *A*_1_ and ${\text{KBq}}/{\text{ml}}$ for *A*_2_ and A_3_, and *L*’s in ${{\text{min}}}^{-1}$ [31]. To create realistic brain tissue time activity curves (TAC), physiologically meaningful ranges for kinetic rate constants previously validated using brain studies were used: *K*_1_ (0.015–0.130) ${\text{ml}}$.${{\text{cm}}}^{-3}$.${{\text{min}}}^{-1}$, *k*_2_ (0.060–0.225) ${{\text{min}}}^{-1}$, *k*_3_ (0.013–0.164) ${{\text{min}}}^{-1}$ and *V*_*b*_ (0.014–0.055) [25]. Note, *K*_*i*_ was computed analytically from *K*_1_, *k*_2_ and *k*_3_. A total of 10 million tissue time activity curves $\left({{\text{kBq}}.{\text{ml}}}^{-1}\right)$ with different Feng function parameters and kinetic rate constants were estimated with the addition of 1D Gaussian noise to the *C*_T_ (t) equation prior to neural network training:

2 $$C_{{\text{T }}} \left( t \right) = \left( {1 - V_{b} } \right).\left[ {\left( {w_{1} e_{1}^{{ - v_{1} t}} + w_{2} e_{2}^{{ - v_{2} t}} } \right) \otimes C_{{\text{P}}} \left( t \right)} \right] + C_{{\text{P}}} \left( t \right).V_{b}$$

3 $${C}_{P}(t)=\left({A}_{1}t-{A}_{2}-{A}_{3}\right){e}^{{L}_{1}t}+ \left({A}_{2}\right){e}^{{L}_{2}t}+ \left({A}_{3}\right){e}^{{L}_{3}t}$$

4 $${K}_{1}= {w}_{1}+ {w}_{2},\quad {k}_{2}= \frac{{w}_{1}{v}_{1}+{w}_{2}{v}_{2}}{{w}_{1}+ {w}_{2}}, {k}_{3}= \frac{{w}_{1}{v}_{2}+{w}_{2}{v}_{1}}{{w}_{1}+ {w}_{2}}- \frac{{{v}_{1}{v}_{2}(w}_{1}+{w}_{2})}{{w}_{1}{v}_{1}+{w}_{2}{v}_{2}}$$

### 1D deconvolution-LSTM

**Fig. 3:**
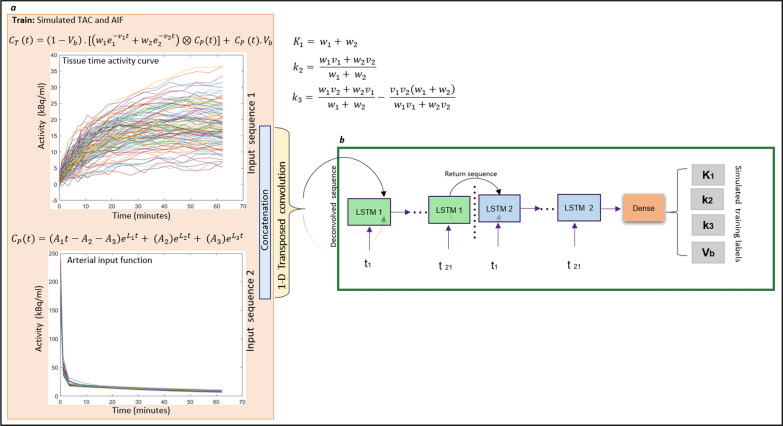
Training the LSTM using simulated tissue activity curves (TAC) and the AIF (*C*_p_) as input (step e in Fig. 1). TAC and *C*_p_ undergo deconvolution using a 1D transposed convolution layer (**a**). Two layers (LSTM 1 and 2) with a fully connected layer (**b**) was trained against simulated training labels

Deep neural networks with fully connected layers are universal function approximators [32]. Estimation of kinetic rate constants given tissue time activity and plasma input function is a nonlinear deconvolution problem in the time domain [*C*_T_ (t) in Eq. 2]. Recently, long short-term memory (LSTM) cells have had wide adoption in sequence-to-sequence prediction problems, while outperforming traditional, recurrent neural networks. This gain has been attributed to the involvement of input, output and forget gates in LSTM cells controlling the essential features to be preserved in the sequence [33]. Our sequence involved the concatenation of the TAC and *C*_*p*_ as the input and deconvolved using 1D transposed convolution layer with filter size of 21 and kernel size of 5, as shown in Fig. 3a. Two LSTM layers (LSTM 1 and 2) of 400 units and finally fully connected layer of size 400 units were used with ReLu activation for all layers (refer to Fig. 3b). The network was implemented upon the TensorFlow using Keras library (version 2.11.0).

### Estimation of plasma input function from histoimages

**Fig. 4:**
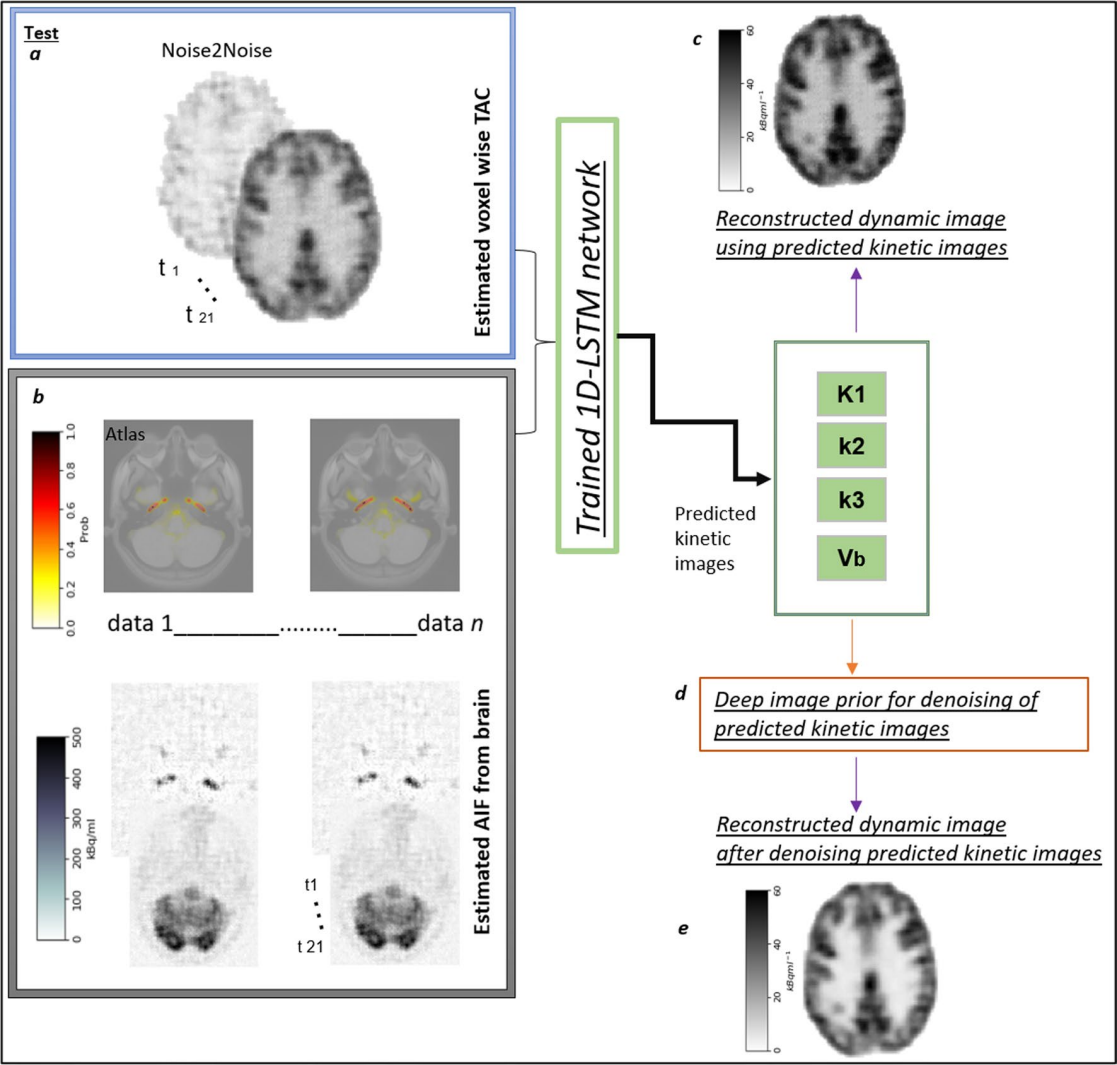
Testing of the LSTM using denoised dynamic images obtained from the **a** Noise2Noise network (at position c in Fig. 1), and using **b** the image derived AIF masked using a vasculature atlas (threshold set at > 0.8) to identify highly probable regions for arterial vasculature. After feeding these as input into the pretrained LSTM kinetic parameters are produced, which are used to reconstruct **c** dynamic images. Parametric images are further processed using **d** an unsupervised deep image prior and **e** denoised version of dynamic images based on having denoised individual parametric images

An arterial brain atlas involving probability of an artery in a voxel was utilized to select the region of higher vascular probabilities in the dynamic brain histoimages after deconvolution and denoising. Figure 4b (top) shows the region with high vasculature probability on the arterial atlas. The statistical arterial brain atlas was created from 544 datasets involving 3D TOF magnetic resonance angiography (MRA) cross-centre images from healthy subjects [34].

The arterial atlas was registered to the last frame of the denoised images at step c in Fig. 1 for each patient, as it had the highest signal-to-noise ratio. The FSL (version 6.0.5.1) package with nonlinear registration was used to map the arterial atlas to the native space of the patient. Finally, dynamic brain images were masked using the atlas using the threshold of > 0.8, resulting in regions of the brain with highly probable arterial vasculature (Fig. 4b, bottom). We used a previously described selection process, based on maximum intensity pixels [35], to estimate the temporal trend in the arterial input function. Ten of the highest pixel intensity temporal profiles were averaged to form the arterial input function [36].

Figure 4a shows the denoised dynamic images from the Noise2Noise network (refer Fig. 2d). Kinetic parameters were estimated pixel-wise using a trained 1D-LSTM model (Fig. 3) and the estimated AIF from brain images using the vascular atlas is shown in Fig. 4b. For validation purposes, dynamic images were generated using the kinetic model and the estimated kinetic parameters, as shown in Fig. 4c. To improve image contrast, the parametric images were next denoised using unsupervised deep image prior [24] (Fig. 4d). Finally, dynamic images were reconstructed using the denoised parametric images (Fig. 4e).

### Image denoising using deep image prior

Parametric images constructed using 1D deconvolution-LSTM netowrk are further denoised using deep image prior. It is an unsupervised deep learning-based method initially developed for image denoising and super resolution. Here a neural network is trained with random noise (*z*) considering noisy image (*x*_*n*_) as label and producing clean image (*x*_*h*_) before overfitting to the noisy image as shown in Eq. (5). The method does not require any additional training data as especially required for in other deep learning methods:

5 $${\theta }{\prime}={{\text{argmin}}}_{\theta } \left| \left|{x}_{n}-f\left(\theta | z\right) \right|\right|,\quad { x}_{h}=f\left(\theta \mathrm{^{\prime}}| z\right),$$

Here, f represents the neural network and theta represents randomly initiated the parameters of network. The deep image prior was used to increase image contrast-to-noise ratio (CNR), thereby increasing visibility of small structures in the parametric images (compare Fig. 4c and e). To prevent from overfitting to image noise, the CNN used in the deep image prior was trained for 700 iterations. The number of iterations was determined to be optimal for the denoising task, achieving a balance between capturing relevant features and avoiding overfitting.

### Image quality metrics and quantitative parameter estimation

The peak signal-to-noise ratio (PSNR), structural similarity (SSIM), and CNR metrics were used for image quality assessment:

6 $${\text{PSNR}}=10{{\text{log}}}_{10} {{\text{max}}}^{2}{\text{MSE}}\left(x,y\right)$$

7 $${\text{SSIM}} \left(x,y\right)=\frac{(2{\mu }_{x}{\mu }_{y}+{C}_{1})(2{\sigma }_{xy}+{C}_{2})}{{(\mu }_{x}^{2}+ {\mu }_{y}^{2}+ {C}_{1}){(\sigma }_{x}^{2}+ {\sigma }_{y}^{2}+ {C}_{2})}$$

8 $${\text{CNR}}= \frac{{\text{Mean}} \left({\text{ROI}}\right)-{\text{Mean}} ({\text{background}})}{{\text{Std}} ({\text{background}})}$$

In Eq. (6), the max corresponds to the pixel of maximum intensity and MSE is the mean squared error between the images estimate image, *x*, and target imaging (PSF-TOF), *y*. SSIM in Eq. (7) involves comparison of luminance, contrast and structural terms, where ${\mu }_{x}$,${\mu }_{y}$ ${\sigma }_{x}$,${\sigma }_{x}$, ${\sigma }_{xy}$ and ${C}_{1-2}$ are the local means, standard deviations, cross-covariance obtained for images *x* and *y*. PSNR and SSIM were calculated to measure image quality at each step with respect to TOF-OSEM. The CNR calculation involved the delineation of a region-of-interest (ROI) and a background region. The background for CNR analysis was taken from the genu and splenium regions of white matter.

For comparison, kinetic parameters (*K*_1_, *k*_2_ and *k*_3_) were estimated by fitting the 2-TCM to the *C*_*T*_ equation in Eq. (1). Fitting was performed using the method of nonlinear least squares without (NLS) and with constraints (NLS-C; *K*_1_/*k*_2_ = 0.7) using the MATLAB-based kinetic modelling simulator (dPETSTEP) [37]. The Patlak method has been widely used with compartment models that contain at least one irreversible compartment [38, 39]. The method yields an estimate of the influx rate under the assumption of the existence of a secular equilibrium. Further, a comparison of the K_i_’s was conducted between those derived through the DL method (Step f in Fig. 1), NLS and the Patlak method.

Note, LSTM was applied to the denoised images at step C of Fig. 1. Patlak, NLS and NLS-C methods were employed on the dynamic images, reconstructed from the scanner, where Patlak derived values were considered as the reference. Additionally, a brain phantom was used with known kinetic parameters to benchmark predictions [40]. While the basis function method has been purported to generally outperform the NLS method, it lacks in tissues predominantly composed of white matter and hypermetabolic grey matter when long scans are used [41]. Due to this reason alone, we benchmarked the performance of our method against the NLS and Patlak methods alone.

### Statistical analysis

The statistical significance of differences in CNR between the parametric images reconstructed using DL and NLS and between the estimated kinetic parameters was evaluated using the paired *t*-test with *p *< 0.05 being used as the threshold for statistical significance. Normality assumptions were confirmed using the Shapiro–Wilks test [42] using a significance level of *p *< 0.05.

The reliability for parameter estimation was assessed by calculating the coefficient of determination (*R*^2^) between estimated kinetic parameters (*K*_1_, *k*_2_ and *k*_3_) and the *K*_*i–*_NLS derived using the NLS method [43]. Further, the statistical significance of *R*^2^ was tested using Fisher's z-transformation to test the null hypothesis that *R*^2^ = 1. In addition, kinetic parameters set in the brain phantom were evaluated with the estimated *K*_1_, *k*_2_ and *k*_3_ using Pearson correlation coefficient (*r*).

## Results

### Comparison of kinetic parameters

**Fig. 5:**
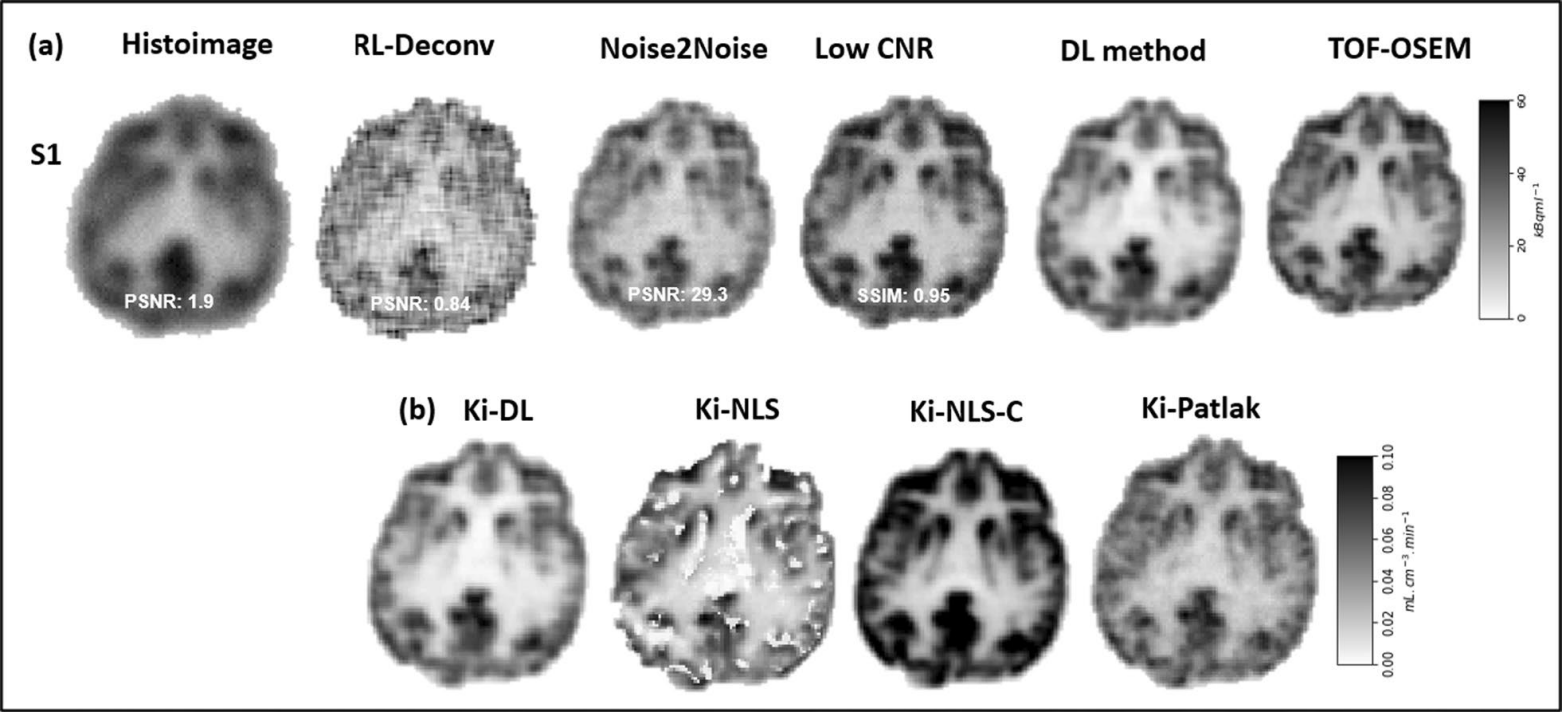
Qualitative and quantitative comparison of results from an example subject. Shown are **a** images generated at steps a, b, c, e’ and g in the reconstruction pipeline (refer to Fig. 1), where PSNR and SSIM were referenced to TOF-OSEM, **b** (left to right) net influx rate constant (*K*_*i*_) reconstructed using the DL method, NLS without and with constraints (*K*_*i*_-NLS and *K*_*i*_-NLS-C) and Patlak method (*K*_*i*_-Patlak)

Figure 5a compares images generated at steps a, b, c, e’ and and g in Fig. 1 for an example subject S1. Histoimages formed the input, RL-Deconv and Noise2Noise refer to the deblurred and denoised images. Label low CNR is the dynamic reconstructed images after the kinetic modelling step (refer to Fig. 4c). Low CNR and DL method outputs are before and after the unsupervised denoising step at d in Fig. 4. TOF-OSEM are the dynamic images generated by the PET scanner. The average PSNR for five subjects from the histoimage to the Noise2Noise (see Additional file 1: Fig. S1) improved from 1.8 to 30, providing high-quality deblurred and denoised images for estimating *C*_*p*_ and *C*_T_.

Figure 5b shows *K*_*i*_ images calculated using the DL, NLS and Patlak methods. Notably, from the comparison, *K*_*i*_-NLS images are noisy then *K*_*i*_-Patlak and *K*_*i*_-DL. The value of the estimated kinetic parameters using the DL and NLS was statistically insignificant for grey (*p *= 0.16, paired t-test) and white matter (*p *= 0.09) and is summarized in Table 2. However, on average, *K*_*i*_ calculated from *K*_1_, *k*_2_ and *k*_3_ obtained using NLS (without and with constrain) for grey matter was found to be at least 35% (0.042/0.027) larger than those estimated using the DL method. When using NLS, a systematic error in any microparameter estimate will lead to bias in Ki, since Ki is analytically computed from microparameters [41]. Average *K*_*i*_ calculated using the Patlak method for grey matter (0.034) is close to the value obtained using the DL method with a 20% (0.034/0.027) overestimation, whereas the NLS method overestimates *K*_*i*_ by at least 24% (0.042/0.034), in comparison with the *K*_*i*_ obtained using Patlak. For white matter, the average Patlak *K*_*i*_ was between the NLS and DL values, with as much as 33% difference. Interestingly, the NLS method greatly overestimates the individual parameters in comparison with the DL method (see Table 2). Moreover, NLS images have noticeably low contrast-to-noise ratios.

**Fig. 6:**
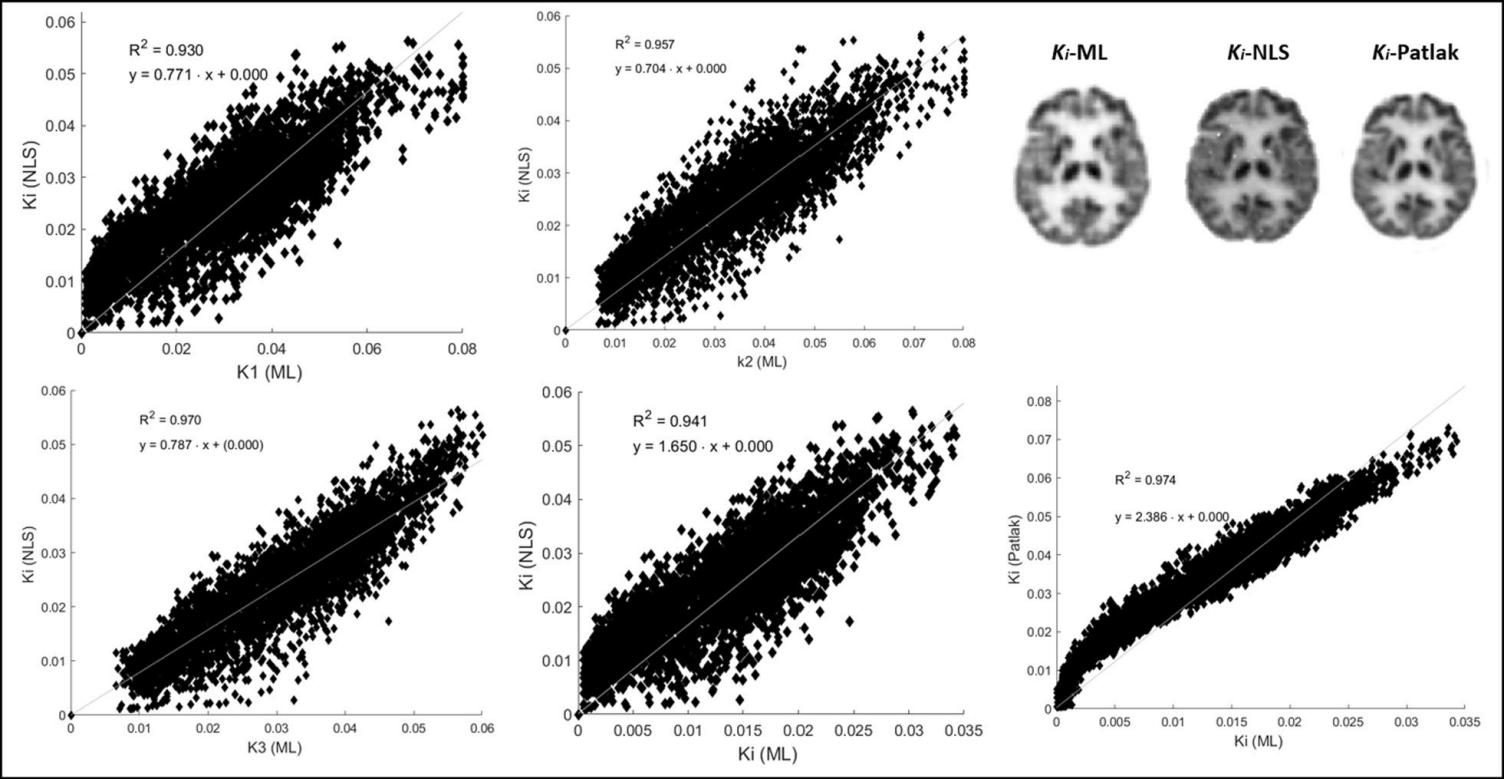
Voxel-wise comparison between the kinetic parameters calculated using the ML method, *K*_*i*_*-NLS* and* K*_*i*_*-*Patlak in Subject 3. Linear regression indicates a linear relationship. *K*_*i*_ values calculated from ML, NLS and Patlak values are shown for an example subject for qualitative comparison

In Table 1, except for S1, the average *R*^2^ is 90%, resulting in a good ability to estimate kinetic parameters. In addition, the *R*^2^ between *K*_1_*, k*_2_*, k*_3_ and *K*_*i*_ and *K*_*i*_*-NLS* were found to be insignificantly different from 1, indicating a strong correlation via Fisher transformation (*p *= 0.07, 0.11, 0.16, 0.10). Figure 6 shows voxel-wise comparison between the kinetic parameters calculated using the ML method, *K*_*i*_-NLS and *K*_*i*_ (Patlak) in Subject 3. Linear regression indicates a linear relationship with tight correlation between *K*_*i*_ (ML) and *K*_*i*_ (Patlak) (*R*^2^ ~ 0.98). Additionally, *K*_*i*_ values calculated from ML, NLS and Patlak values are shown for an example subject for the purpose of qualitative comparison. Further, to validate findings, kinetic parameters set in the brain phantom correlated strongly with the estimated *K*_1_*, k*_2_* and k*_3_ (*r *= 0.91, 0.92 and 0.93) with a mean squared error of less than 0.0004.

**Table 1:** Coefficient of determination (*R*^2^) is provided for DL benchmarked against the NLS method for subjects S1–S5

Subject	Coefficient of determination (* R*^2^)			
	*K*_1_	*k*_2_	*k*_3_	*K*_*i*_
S1	0.75	0.80	0.85	0.79
S2	0.89	0.91	0.93	0.91
S3	0.93	0.95	0.97	0.94
S4	0.94	0.96	0.96	0.96
S5	0.82	0.86	0.89	0.84

### Comparison of image quality

**Fig. 7:**
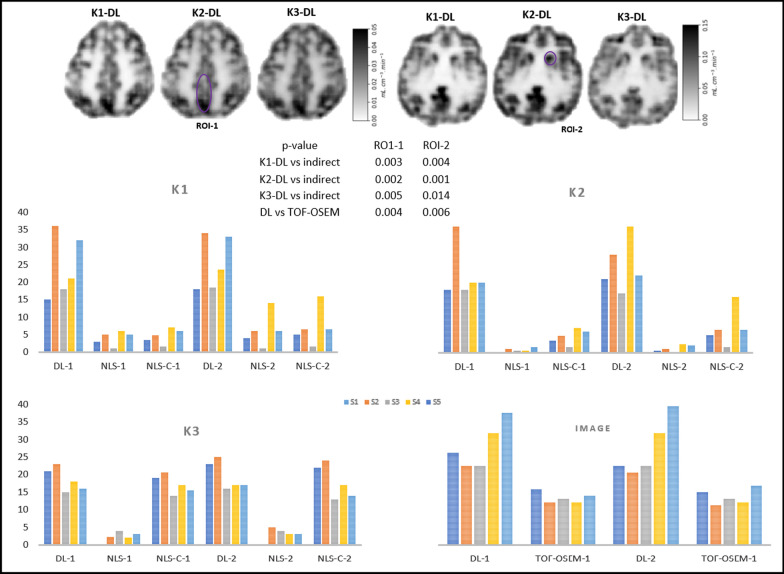
Illustration of reconstructed parametric images (*K*_1_, *k*_2_ and *k*_3_) by the DL method (top row). Comparison of the CNR of individual kinetic parameters (between DL, NLS and NLS-C) and last dynamic timeframe (between DL and TOF-OSEM) for the two ROIs (bottom bar charts)

The difference between *K*_*i*_ images is due to improved contrast in *K*_*i*_-DL images as reported in Fig. 5b. ROIs 1 and 2 both in grey matter were considered for CNR analysis of *K*_1_, *k*_2_ and *k*_3_ maps (see Fig. 7), along with the last time point dynamic images. The bar graphs depict the CNR calculated from the DL and NLS method produced parameters for both ROIs. The DL method significantly outperforms (*p *< 0.05 for all kinetic parameters in two circled ROIs, see Fig. 7) the NLS method. The level of CNR improvement over NLS varies with subject and ROI. In addition, to reconstruct parametric images for a 2D slice (256 × 256 image size in MATLAB with 32 GB RAM on 64-bit OS), conventional NLS fitting techniques typically require ~ 140 min, whereas the proposed method required only 45 min (reducing the time of reconstruction by 69%). Most of the time was taken by the DIP step (~ 43 min).

Further, the average SSIM achieved for the reconstructed dynamic images from the predicted kinetic parameters with respect to TOF-OSEM was found to be greater than 0.94 (low SNR image, Fig. 5a). This finding validates that the DL approach can preserve information in the dynamic images, while at the same time improving the quality of these images.

## Discussion

Parametric images can provide extensive information for certain clinical applications in ^18^F-FDG-PET imaging. However, there are distinct challenges associated with producing high-quality parametric images, which can potentially be addressed using novel data-driven approaches. We proposed a deep learning framework for direct PET parametric image reconstruction that achieves significant improvements in the quality of estimated PET microparameters. The findings were validated using an assessment of parametric map image quality and linear regression against benchmark values. Importantly, our proposed framework for PET parametric image reconstruction does not require invasive procedures for arterial blood sampling or the incorporation of information from other imaging modalities.

We found good correspondence between the reconstructed microparameter images (*K*_1_, *k*_2_ and *k*_3_) obtained using our proposed new method and those obtained from indirect NLS (with and without constraints, see Table 2 and Fig. 6), as well as the macroparameter, *K*_*i*_, produced using the Patlak graphical method. The scatter plots between *K*_*i*_-NLS and ML-based *K*_1_, *k*_2_ and *k*_3_ values are used to identify voxel-wise relationships and assert regions sensitive to high noise and low signal (regions of white matter from *K*_*i*_-NLS images can be seen in Fig. 6). We observed a highest positive correlation between the *k*_3_-ML and *K*_*i*_-NLS and relatively tight and significant correlation between *K*_i_-ML and Patlak *K*_i_ values.

**Table 2:** Comparison of kinetic parameters in grey and white matter estimated using DL and the NLS, NLS-C and Patlak methods

Kinetic parameter	Deep learning		NLS		NLS-C		Patlak	
	GM	WM	GM	WM	GM	WM	GM	WM
*K1*	0.043–0.087	0.014–0.020	0.168–0.250	0.083–0.136	0.174–0.280	0.0913–0.151	–	–
*k2*	0.049–0.095	0.012–0.019	0.086–0.171	0.048–0.169	0.095–0.201	0.052–0.176	–	–
*k3*	0.043–0.059	0.016–0.020	0.026–0.040	0.016–0.023	0.047–0.090	0.024–0.031	–	–
*K*_*i*_	0.021–0.033	0.008–0.010	0.030–0.110	0.034–0.067	0.055–0.130	0.041–0.077	0.021–0.046	0.008–0.016
*p values*	–	–	0.16	0.06	0.17	0.09	–	–

Values provided are based on 95% confidence across all subjects. The *p* values were calculated to establish significant mean differences between *K*_1_, *k*_2_, *k*_3_ between the DL and NLS results (with and without constraints)

The spread observed in Fig. 6 can be attributed to the regions of white matter, which are prone to high noise, particularly in *k*_2_ images reflecting rates of tracer phosphorylation that are used to calculate *K*_*i*_-NLS. Substantial bias between *K*_*i*_-Patlak and *K*_*i*_-ML has been noted in connection with the assumed time for pseudo-equilibrium in Patlak analysis [40]. Additionally, the bias between *Ki*-NLS and *Ki*-ML may be influenced by hypermetabolic grey matter region, aligning with findings from previous studies [25, 41, 44].

Our method is the first of its kind to use histoimages directly, as such making any comparison with projection-based direct reconstruction methods is ambiguous for interpretation. Therefore, we compared it with existing indirect methods to establish image quality. In addition, while not reported here, we tested the trained LSTM network on the standard dynamic PET images generated by the scanner and similar parameters were estimated (see Additional file 1: Fig. S2). A notable advantage of using histoimages, however, is that the standard PET image reconstruction step does not have to performed. The results from both simulation and real data demonstrate that our proposed framework can lead to high-quality parametric images of PET kinetic parameters, which may pave the way forward towards routine ^18^F-FDG-PET parametric mapping in the clinic.

### Image-to-image parametric mapping

Our outlined deep learning method represents a significant advancement for the field as it uses histoimages as the input for direct PET parametric image reconstruction. Unlike sinograms, histoimages are more amenable to machine learning algorithms and allow for near-real-time 3D image reconstruction. By storing the time-of-flight information in the histoimages, interpolation and ray-tracing operations required in forward and back-projection can be avoided and their impact on image resolution is minimized [20]. Recently, several methods have adopted TOF information to convert coincidence events into a histoimage format, which was then used as input for machine learning frameworks (i.e. in the CNN context) to reconstruct the final images [19, 20, 45]. However, these approaches are limited to image reconstruction tasks while also requiring labelled training and testing data.

Histoimages obtained from TOF-PET scanners are susceptible to blurring along the TOF dimension. The task of deblurring histoimages can be categorized as either a blind or non-blind deblurring method. Non-blind deblurring methods require both the blurry image and the blur kernel as inputs to estimate a clean version of the image [29]. Blind deblurring of images is a more challenging problem, taking only the blurry image as input before a clean image is generated [29]. Since the blurring kernel in TOF-PET can be estimated, we opted to use the non-blind deblurring of images approach. We implemented iterative [26] and non-iterative methods [27], as it was unclear which method may best suit this application. We found the non-iterative implementation to be faster in execution than the iterative version. However, it lacked the image quality achieved using the iterative method (not reported in the results). This was particularly evident with noisy images, as is the case for PET histoimages degraded by Poisson noise.

### Training and testing data

A key advantage of our deep learning method is that it does not require paired clinical data. Supervised machine learning methods typically rely on a large number of paired images (i.e. clean target and noisy counterparts) [1]. The collection and acquisition of a large training dataset is often impractical, especially in the case of PET imaging where radiotracers are administered to healthy volunteers raising ethical concerns. In recent years, unsupervised deep learning methods have emerged as a potential solution for reducing computational resources and execution times while improving the ability to model noise, which is particularly important for direct PET parametric image reconstruction. However, the application of these methods have been limited to macro-parametric images such as those used in Patlak or Logan type analyses.

The deep learning framework was trained using simulated histoimages and five oncology subjects were used for validation, which is consistent with recently published studies using only four subjects [10]. Recent studies have also used simulated PET histoimages to account for photon arrival times and a Gaussian kernel for the probability of photon pairs along the line of response [19, 45]. TOF-based image reconstruction incorporated as part of the OSEM method essentially modifies the entries of system matrix with TOF kernel-weighted operations along the lines of response. As such, TOF information is inherently captured in our deep learning-based parametric image reconstruction and, also in images generated using TOF-OSEM reconstruction prior to generating parametric maps of kinetic parameters using traditional approaches.

### Computational efficacy

Our deep learning inspired method offers a significant computational speed advantage over conventional methods for the estimation of parametric images without the need for extensive computing resources. Indirect methods of producing parametric images are time-consuming and generate images with low SNR [46]. Direct methods involve kinetic modelling during the iterative image reconstruction process, and they are computationally expensive than indirect methods leading to extensive reconstruction times [16]. Moreover, direct methods may require offline reconstruction, which can limit their practical use in clinical settings [2]. We were able to achieve a 69% reduction in parametric map generation time compared to the conventional nonlinear least squares method. This can further be decreased, potentially by as much as 97%, by implementing the deep learning framework on a GPU system [47].

### Limitations

One drawback of the iterative Richardson-Lucy algorithm is that, in the presence of noise in images, the deconvolution process tends to converge to a solution heavily influenced by the noise. To overcome this issue, we empirically determined that early stopping at 30 epochs (which took less than 5 s per slice for 21 frames in MATLAB without GPUs) using the scanner-dependent blurring kernel yielded satisfactory deblurred images. To mitigate noise in the deblurred images, two options were considered. The first option was to employ prefiltering techniques, and the second option was to introduce regularization terms. Due to the need for careful tuning of individual hyperparameters and inherently excessive execution times, we did not employ regularization terms in our approach. Instead, a neural network-based (Noise2Noise) approach trained on an atlas for denoising was implemented [23].

Our study provides proof of concept for the application of histoimages for kinetic parameter estimation. In this work, we were unable to use scanner generated histoimages as such software tools are not generally available for Siemens scanners (the Bern centre has an evaluation package available at the moment). Future research will evaluate scanner obtained histoimages. In addition, the use of simulated histoimages prevents evaluation of proposed method against corrections required for attenuation, scatter, and random coincidence events. A previous deep learning method used real histoimages with attenuation maps as inputs to neural networks for image reconstruction [20]. This type of approach could be considered in the future to explore the potential of machine learning to perform autonomous corrections in parameter estimation. It may also resolve the bias observed in Fig. 6, where *Ki*-ML is underestimated with respect to *Ki*-Patlak and *Ki*-NLS. Further, the parametric images were generated by an LSTM network trained using simulated TACs, assuming a standard two-tissue model with three rate constants and the Feng function was employed to generate input functions for these simulated curves. The LSTM network may be sensitive to the biological range of simulated parameters used in training and to the rate at which FDG is infused. In particular, variations between slow bolus and fast bolus administrations are known to affect the results [48]. Future validation may be needed to assess method robustness.

## Conclusion

We proposed a deep learning-based reconstruction method for parametric imaging in PET using histoimages. We produced parametric images of higher quality than those obtained using the conventional, nonlinear least squares method. The new method is non-invasive and does not require invasive arterial input function estimation. As such, it can be applied to subject-specific dynamic PET data alone. The proposed method highlights the potential future opportunities for deep learning in parametric imaging.

## Supplementary Information

**Additional file 1: Fig. S1.** Qualitative and quantitative comparison of results from five subjects. **Fig. S2.** Regression plots illustrate a comparison of kinetic parameters derived from histoimages using our proposed pipeline (in Fig. 1) and those obtained directly through a trained LSTM network from standard dynamic images. The yellow box specifically compares the estimated Ki images from both these methods.

## Data Availability

The data that support the findings of this study are available from [25] but restrictions apply to the availability of these data, which were used under license for the current study, and so are not publicly available.

## Abbreviations

PET: Positron emission tomography
TOF: Time of flight
DL: Deep learning
SNR: Signal-to-noise ratio
CNR: Contrast-to-noise ratio
NLS: Nonlinear least squares
^18^F-FDG: ^18^F-fluorodeoxyglucose
