# Supplementary material for: ParaPET: non-invasive deep learning method for direct parametric brain PET reconstruction using histoimages
Source: EJNMMI Res. 2024 Jan 30;14:10. doi: 10.1186/s13550-024-01072-y (PMC11374951; doi:10.1186/s13550-024-01072-y)
Supplement: Supplementary file 1 — Additional file 1: Fig. S1. Qualitative and quantitative comparison of results from five subjects. Fig. S2. Regression plots illustrate a comparison of kinetic parameters derived from histoimages using our proposed pipeline (in Fig. 1) and those obtained directly through a trained LSTM network from standard dynamic images. The yellow box specifically compares the estimated Ki images from both these methods. [file 13550_2024_1072_MOESM1_ESM.docx]

**Supplementary figures:**


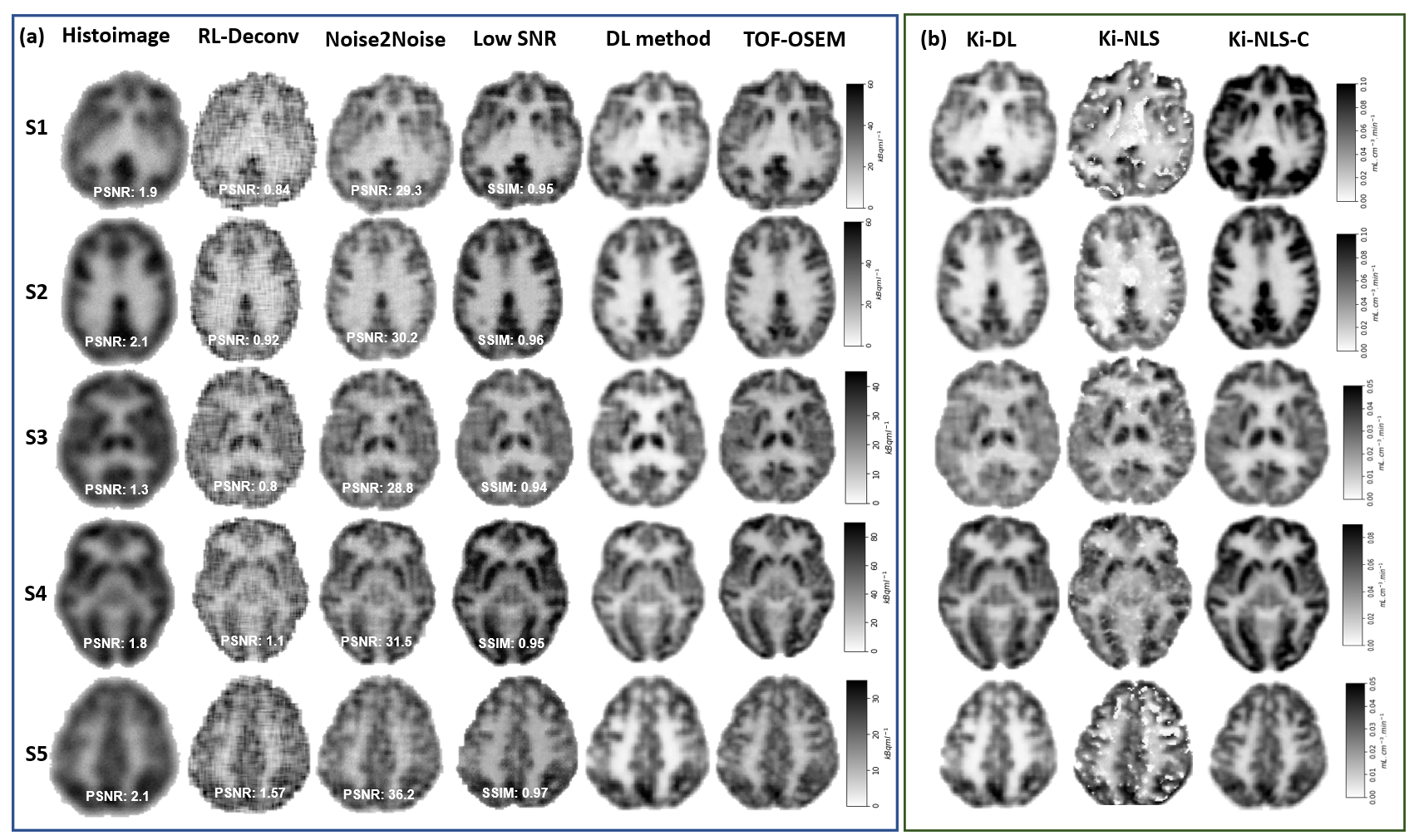


Figure 1s: Qualitative and quantitative comparison of results from five subjects.

**
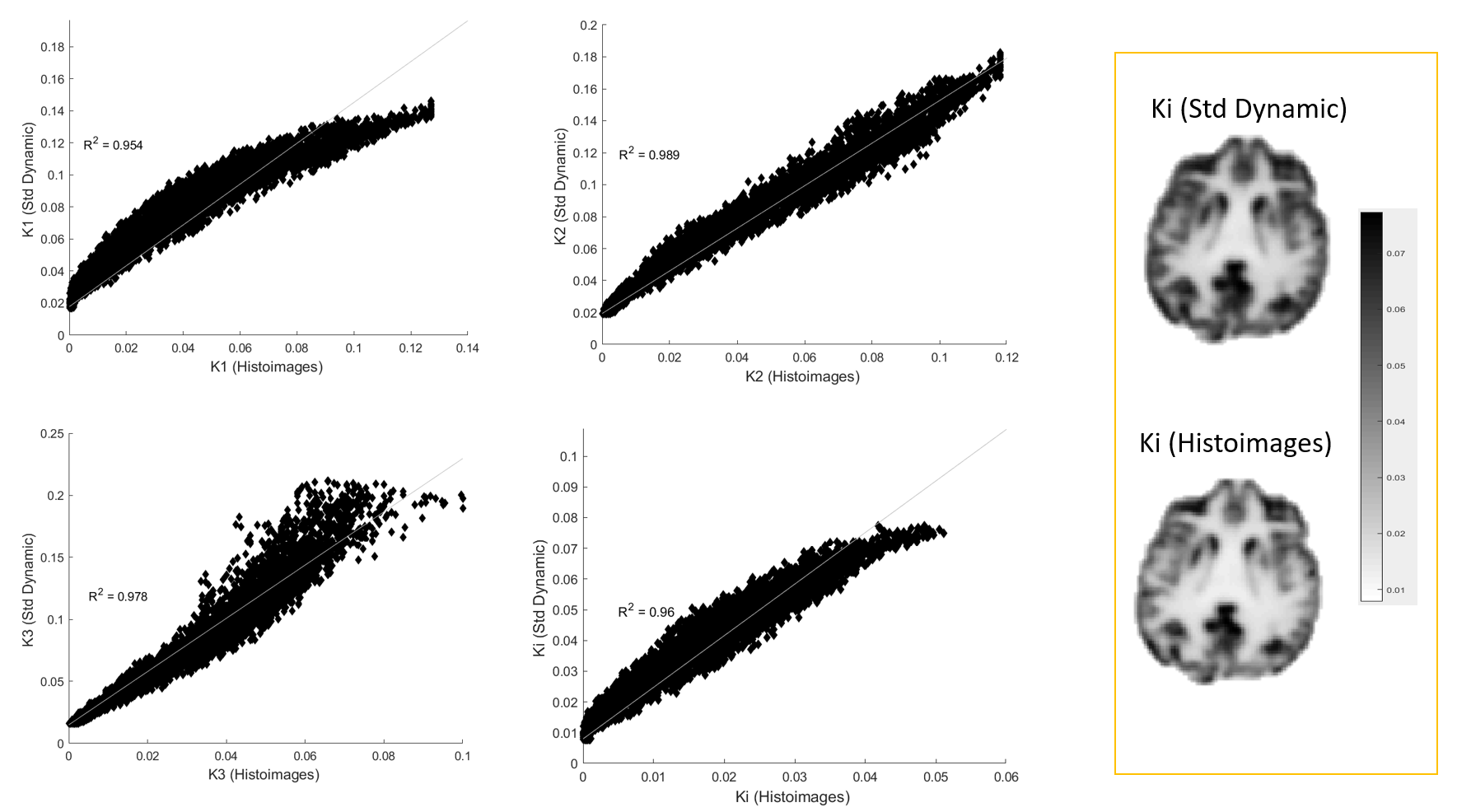
**

Figure 2s: Regression plots illustrate a comparison of kinetic parameters derived from histoimages using our proposed pipeline (in Figure 1) and those obtained directly through a trained LSTM network from standard dynamic images. The yellow box specifically compares the estimated *Ki* images from both these methods.
